# The Anti-leishmanial Efficacy of *Artemisia dracunculus* Ethanolic Extract in Vitro and Its Effects on IFN-γ and IL-4 Response

**Published:** 2017

**Authors:** Reza REZAEI, Khosrow HAZRATI TAPPEH, Shahram SEYYEDI, Peyman MIKAILI

**Affiliations:** 1. Dept. of Parasitology & Mycology, School of Medicine, Urmia University of Medical Sciences, Urmia, Iran; 2. Cellular and Molecular Research Center, Urmia University of Medical Sciences, Urmia, Iran; 3. Dept. of Immunology, School of Medicine, Urmia University of Medical Sciences, Urmia, Iran; 4. Dept. of Pharmacology, School of Medicine, Urmia University of Medical Sciences, Urmia, Iran

**Keywords:** *Leishmania major*, Promastigote, HPBMCs, IFN-γ, IL-4, *Artemisia dracunculus*

## Abstract

**Background:**

Leishmaniasis is a parasitic disease that appears with a range of symptoms including cutaneous, mucocutaneous, and visceral leishmaniasis. The present study sought to determine the antileishmanial effect of the extract of *Artemisia dracunculus* (Tarragon) compared to control treatment with pentavalent antimony (meglumine).

**Methods:**

This experimental study was performed in 2014–2015. *A. dracunculus* were collected from West Azerbaijan Province, Iran and dried; then the ethanolic extract of the plant was prepared. The effect of different concentrations of Artemisia’s extract was compared with Glucantime ® in the stationary phase by MTT colorimetric assay and Trypan blue staining. Human peripheral blood mononuclear cells (HPBMCs) treated with *L. major* and production of IFN-γ and IL-4 cytokines measured at concentrations of 25, 20, 10 and 5μg/ml *A. dracunculus*.

**Results:**

Treatment with the extract did not affect the survival of the parasites during the first 48 h; however, on the third day (72 h), all concentrations significantly reduced the number of parasites with an efficacy of more than 50% at 10 μg/ml (*P*<0.01), 20μg/ml (*P*<0.001), and 25 μg/ml (*P*<0.0001). Moreover, IFN-γ and IL-4 secretion from the HPBMCs was significantly affected in a dose-dependent manner, compared to the control (no extract). The IFN-γ/IL-4 ratio further confirmed this notion.

**Conclusion:**

*A. dracunculus* extract cannot only exert potent antileishmanial activity but may also enhance cellular immunity to this parasite. Further studies are required to determine the main compound(s) responsible for these effects of the plant.

## Introduction

Leishmaniasis is a common disease of humans and animals and is found all over the world. The disease is caused by a protozoan called *Leishmania major* (*L. major*). The parasite lives in mononuclear phagocytic cells of vertebrates. In general, different clinical forms of the disease can be divided into two categories: visceral leishmaniasis (locally known as kala-azar) and cutaneous leishmaniasis. Visceral leishmaniasis (VL) is a serious disease and most suffered patients will die if they are left untreated. Cutaneous leishmaniasis (CL) has mainly spontaneous desire to improve; however, due to the remaining scars, it has social and psychological consequences (1). The data on all types of cutaneous leishmaniasis in Iran were collected from 1983–2012. Over this period the total number of cases reached 566532 were cutaneous leishmaniasis. Annual average incidence of CL was 88418 of 32 per 100000 inhabitants over the 30-yr period (2). Therefore, annual VL incidence in Iran was 0.449 cases/100000 inhabitants during last decade (3).

Various chemical drugs are used to treat leishmaniasis among which pentavalent antimony compounds, antimalarial drugs, and antibiotics can be mentioned. In addition to economic damage inflicted on families, due to the repeated injections of high doses, the drugs lead to side effects such as liver, heart and biochemical disorders. In addition, these compounds have limitations such as the lack of efficacy through oral administration, the length of treatment period, and weak response to treatment in about 15%–10% of the cases. The extract of *Artemisia dracunculus* (*A. dracunculus*) namely tarragon, from the Asteraceae family (locally known as Marki), which mainly contains artemisinin compounds, have anti-parasitic effects due to its endoperoxide groups. In addition to the mentioned compounds, it contains alkaloids, glycosides, essential oils and flavonoids, which possess insecticidal, antifungal, antithrombotic, anti-hyperlipidemia, and anti-tumor properties (2). The extract of this plant is effective in the treatment of cutaneous lesions of cutaneous leishmaniasis in mice (3). However, research shows no human studies in this area. Moreover, this effect of the plant extract on macrophages and its effect on the immune system response, which plays an important role in the protection against the propagation of *L. major*, has not been considered yet. The plant has some effects on the function of immune cells such as macrophages.

In this study for the first time, the effect of the hydroalcoholic extract of *A. dracunculus* on the proliferation of *L. major* in the cell culture of peripheral blood mononuclear cells (PBMCs) derived from human blood. Moreover, its impact on the production of the cytokines IFN-γ and IL-4 and immune system variations between TH_1_ and TH_2_ responses was evaluated.

## Materials and Methods

### Extraction of the Ethanolic extract of *A. dracunculus*

*A. dracunculus* was planted and collected in the Village Baghcheh-Jik, Salmas city, West Azerbaijan Province, Iran during 2014–2015. After botanical confirmation in the Department of Pharmacology, Urmia University of Medical Sciences and the Department of Botany, Faculty of Science, University of Urmia; it received a herbarium code (Urmia Herb Number # 7568) and a voucher was deposited. The plant was dried in the shade at 35–40 °C and was ground into a fine powder. To produce the hydroalcoholic extract, 200 gr of the plant powder was soaked into 800 ml of 70% ethanol 70 prepared with deionized distilled water for 3 d and the solution was shaken once every 24 h and then filtered through a Whatman‘s paper number one. Removing the alcohol from the extract solution was performed by evaporation in a vacuum using a rotary device until a liquid gel was formed. With this method, the hydroalcoholic extract of the plant was prepared, then the efficiency of extraction was determined and different concentrations of the extract were prepared as required.

### The preparation and cultivation of the standard strain of *L. major*

Promastigotes of the standard strain of *L. major* (MRHO/IR/75/ER), prepared by the Department of Parasitology & Mycology, Tehran University of Medical Sciences, were used. First, the promastigotes of *L. major* were cultured in a medium of (biosera) 1640-RPMI containing 10% fetal calf serum, 100 IU/ml of penicillin and 100 IU/ml of streptomycin were cultured at 2 ± 24 °C. Then, the promastigotes, which had reached the stationary phase in the medium, were counted using a hemocytometer under the light microscope and were modified to 1×10^6^ cells.

### Addition of the promastigotes and the dilution of the extract into plates

About 200 ml of the culture medium containing promastigotes of *L. major* in the stationary phase of growth were added to each well of a 96-well plate in duplicate form so that in each well, 10^6^ promastigotes were present. Then, concentrations of 1, 5, 10, 20, and 25 μg/ml of the extract were prepared and 20 ml was added to 96-well plate in duplicate. Moreover, a control (blank) of culture medium without promastigotes or the extract, a positive control (wells containing promastigotes and meglumine [Glucantime]) and a negative control (wells containing promastigotes only) was considered. Then the plate was incubated at 24 °C for 72 h. The counting was performed with intervals of 24, 48, and 72 h by trypan blue using a Neubauer slide. Results were expressed as the concentration that inhibited parasite growth by 50% (IC50: half-maximal inhibitory concentration).

### Preparation of human peripheral blood mononuclear cells (HPBMCs)

First, in a sterile falcon, the same volumes of sterile PBS and whole blood were mixed together. Then, in another ficoll-containing sterile falcon, one third of the mixture was gently added. Then it was centrifuged for 20 min at room temperature (25°C) at 2000 rpm. Then, the buffy coat was carefully removed and was poured in another sterile tube. These cells were washed twice, each time with 10 ml of sterile RPMI. To complete the process, centrifugation was performed at a temperature of 25°C for 5 min at 2000 rpm and then one ml of RPMI medium was added to the cells.

### The exposure of HPBMCs with standard strain of *L. major*

First, HPBMCs were cultured in sterile conditions inside RPMI medium containing flasks and then incubated for 4 h at 37 °C in the presence of 5% CO_2_. Next, the RPMI was removed and the remaining cells were gently washed twice with sterile PBS so that non-adherent cells could be obtained. After washing, RPMI was added to the flask and the cells were evaluated under an inverted microscope for the presence of HPBMCs. After confirming the existence of HPBMCs, the culture flask was placed in an ice container to remove HPBMCs. After counting of the cells, each HPBMC was exposed to four cells of *L. major* in the stationary phase. The culture flask was incubated at 37 °C in the presence of 5% CO_2_ for 4 to 24 h to let the parasites enter the mononuclear cells. The cells were gently washed twice with PBS to remove the free cells. RPMI medium containing 10% FBS was added to the flask and was placed on an ice container to uproot the parasite-containing HPBMCs. Finally, slides were prepared and studied with Giemsa staining.

### The exposure of *L. major* infected HPBMCs with different concentrations of the hydroalcoholic extract of *A. dracuncultus*

First, 10^6^ HPBMCs were added to each well of a 24-well culture plate and were incubated at 37 °C for 3 h in the presence of 5% CO_2_. The non-adherent cells were removed by washing and the cells were exposed to the parasites in the stationary phase at a ratio of 1 to 4. After 4 h, the cells were washed and the free parasites were removed. Infected HPBMCs evaluated in three groups including 1, 5, 10, 20, 25 mg/ml of ethanolic extract of *A. dracunculus*, positive control treated by Glucantime® and negative control without any treatment. About 1990 μl of RPMI containing FBS 10% and 10 μl of different concentrations of the hydroalcoholic extract of *A. dracuncultus* at a dilution of 1, 5, 10, 20, 25 mg/ml were added to the wells. The plates were incubated at 37 °C in the presence of CO_2_. On the second and third days, different concentrations of the extracts were prepared and then fixed with methanol and stained with Giemsa to count the number of parasites in 100 mononuclear cells to obtain the IC50 of the extract.

Investigating the effect of hydroalcoholic extract of *A. dracuncultus* on the proliferation of *L. major* in HPBMCs and on the immune system (the shift between TH_1_ and TH_2_ as indicated by IFN-γ and IL-4 response), While the HPBMCs were exposed to *L. major* at a ratio of 1 to 4 cells in 24-well plates, after 24 h, the supernatant was removed to check the cytokines IFN-γ and IL-4 determined by ELISA reader. Then, different concentrations of the hydroalcoholic extract of *A. dracuncultus* were added to the plates and were incubated for 72 h at 37 °C with 5% CO_2_. The supernatants were collected and the concentrations of the cytokines were measured by ELISA to compare the production of cytokines such as IFN-γ and IL-4 before and after treatment with different concentrations of the extract.

## Results

**Fig. 1:**
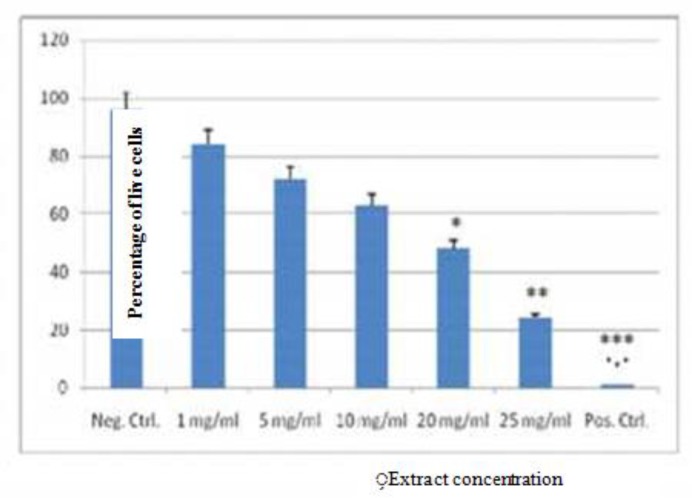
The anti-Leishmanial effect of 1, 5, 10, 20 and 25 mg/ml of the hydroalcoholic extract of *A. dracunculus* on the growth of promastigotes (all the numerical values are multiplied by 10^6^). *P<0.05;** *P*<0.001;*** *P*<0.0001

The effect of different concentrations of the extract of *A. dracunculus* on the proliferation of promastigote forms of the parasite in culture is presented in Fig. 1. In the present study, the effects of different concentrations of tarragon on the growth of promastigotes cultured in RPMI in the stationary phase in three consecutive days were compared with a concentration of 4 mg/ml meglumine (as the positive control) and promastigotes in RPMI without treatment (as the negative control). On the third day (72-h), compared to 4 mg/ml meglumine, the concentrations of 10, 20, and 25 mg/ml of the extract showed significant anti-leishmanial activity (*P*<0.01, *P*<0.001, and *P*<0.0001, respectively) (Fig. 1).

**Fig. 2:**
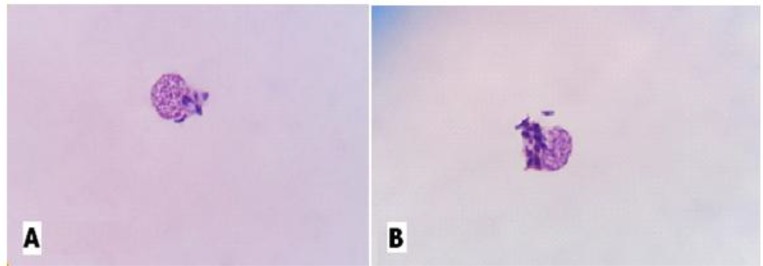
HPBMCs (A, B) infected with *L. major* amastigotes (Giemsa staining, magnification ×100)

The effects of different concentrations of hydroalcholic tarragon on the number of amastigotes of *L. major* in HPBMCs after 48 h and 72 h, was also evaluated compared with negative controls (without extract), which showed a significant reduction in the percentage of contaminated cells after 48 h (*P*>0.05) (Fig. 2). Moreover, after 72 h, it showed a significant reduction in the infection rates at dilutions of 20 and 25 mg/ml of the extract, compared to the negative control (*P*<0.0001). Fig. 2 compares the percentage and number of parasites in HPBMCs without the extract and those exposed to different concentrations of the extract.

**Fig. 3:**
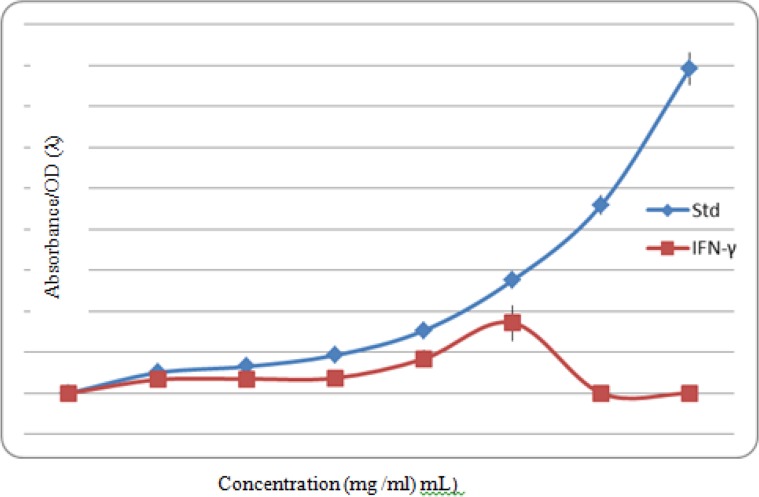
Comparison of the generation of IFN-γ by HPBMCs in culture media with or without the extracts of *A. dracunculus*

With regard to the amount of IFN-γ secreted by HPBMCs in the culture media containing plant extracts, compared with pre-exposure and post-exposure extract, especially at doses of 20 and 25 mg/mL, the difference of observed optical absorption was statistically significant (*P*<0.0001) in a way that with an increase in the concentration, the optical density increased gradually (Fig. 3).

**Fig. 4:**
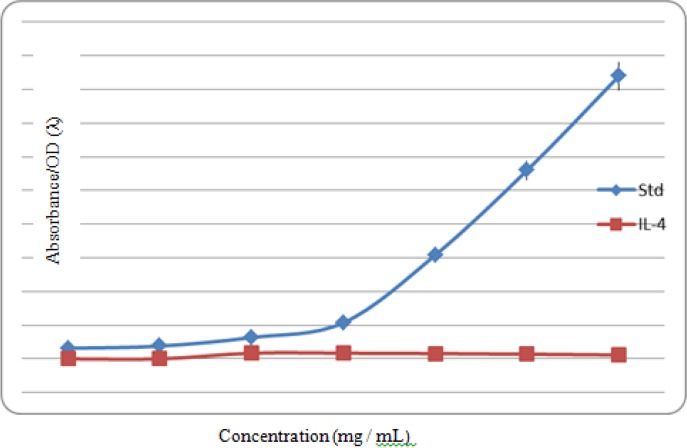
Comparison of IL-4 secretion by PBMCs in culture media with and without treatment with the extract of *A. dracunculus*

The amount of IL-4 secreted by HPBMCs in culture media was significantly different between pre-exposure and post-exposure to the extract, especially at doses of 20 and 25 mg/ml (*P*<0.0001) (Fig. 4).

## Discussion

Different ways including direct counting of living cells using homosignometer slides under the microscope, measuring enzyme activity and colorimetric methods (Colorimetry) based on tetrazolium salt reduction reaction, have been introduced to study the growth and viability of Leishmania promastigotes (4). Compared to the direct counting method, which is a time to consume, and at the same time difficult method, colorimetric methods have many advantages i.e. colorimetric methods are easy, inexpensive, sensitive, reliable, reproducible and due to the lack of radioisotope material, these methods are reliable (5).

Accordingly, in this study, the direct counting and colorimetric methods were applied to compare the activity of different concentrations of the extract of tarragon on *L. major* and Glucantime® was used as the control. By increasing the concentration of tarragon and Glucantime® as the positive control, the inhibitory effect on *L. major* promastigotes increases. In the present study, the comparison of the mean absorbance of different concentrations of the extract of tarragon and the control drug was significantly different which shows that all different concentrations of the extract ratios of tarragon can decrease the growth of *L. major*.

The results of direct counting and colorimetric method using MTT showed that on neither the first nor the second day (24/48 h) the concentrations of tarragon extract did not exert any significant effects. However, by the lapse of time i.e. on the third day (72-h), the concentrations of 5, 10, 20 and 25 mg/ml of the hydroalcoholic extract of tarragon were shown to be efficient with a level of significance of (*P*<0.01) for 10 mg/ml, (*P*<0.001) for 20 mg/ml, and (*P*<0.0001) for 25 mg/ml. More than 50% of the parasites were removed compared to 4 mg meglumine that resulted in the complete removal of the parasites. Among 7 studied extracts, *A. dracuncultus* resulted in better treatment of small wounds and the complete removal of Leishman bodies from the lesion and prevented the expansion of large wounds (by reducing the number of parasites) (3). In an in vitro study on the anti-leishmanial effects of a number of Artemisia species, almost all eleven species of Artemisia in Iran had effects against *L. major* promastigotes (6). The aqueous extract of the leaves of *Artemisia Indica* with an IC50 of 430 μg/ml had anti-lishmanial effects (7). In the study on the anti-leishmaniasis effects of different species of *Artemisia*, it became evident that some species have more potent antiparasitic properties. However, all species of Artemisia have anti-leishmanial effects. The ethanol extract of Artemisia species *A. kulbadica* and *A. ciniformis* with an IC50 of 25 μg/ml have shown the greatest anti-Leishmanial effects (8). In a study on the effects of non-polar ethyl acetate extract of *A. frgrans*, this type of extracts has limited effects on promastigotes of *L. major* (9). Ethyl acetate extracts from Artemisia species (species except for *A. turanica* and *A. fragrans*) in dichloromethane extracts were more effective (10). In addition, in comparison with other studied Artemisia extracts, hexane extracts were less effective against *L. major*. The hexane extracts of tarragon and a few other species had minimal impact, including species of *A. annua*, *A. fragrans*, *A. turanica*, *A. absinthium* and *A. biennis*. The low impact of hexane extracts of these plants on the genus Leishmania compared to other types of extracts (11). Numerous phytochemical screening has so far identified active ingredients in many Artemisia species, including monoterpenes, terpenes, monoterpenes, terpene lactones, flavonoids, coumarin, sterols, and polyacetylenes (12). Now, little information is available, on which compounds possess more anti-leishmanial effects. Nevertheless, what is certain that the ethanol extract of Artemisia species has leishmanicidal effects, and that the anti-leishmanicidal effect of these preparations on *L. major* is dose-dependent (13). Since the major compounds present in this plant are artemisinin’s, the potent antileishmanial activity of the extract might be at least partially attributed to the presence of such compounds. Further research must still be conducted to properly identify the natural anti-leishmaniasis compounds or fractions of active ingredients in the plants. However, in this study, the cytotoxic effect of the hydroalcoholic extract of tarragon on the genus Leishmanial is dose-dependent. Various Ethiopian species such as *A. absinthium* and other species (*A. aethiopica* and *A. donovani*) can have bacteriostatic properties on a variety of strains of *L. aethiopica* and *L. donovani* and especially on promastigotes and amastigote forms (14). Therefore, pharmaceutical forms of plant seeds have been produced and used (15). Apart from anti-Leishmanial effects, oily extract of *A. absinthium* has been used to treat infections of eleven bacterial pathogens (16). About 200 μg/ml of the extract of *A. tincturia* can significantly inhibit the growth of Leishmania species. However, the extract of this plant can be mixed with the extract of *Peganum harmala*. In this combined form, its antileishmanial effect can be more specific on the amastigotes (17). The results of this study show the significant reduction of amastigotes in effective doses of tarragon (*P*<0.05). The effect of different extracts of Artemisia (including *A. absinthium* and *A. vulgaris*) on *L. amazonensis* and its toxicity in mice peritoneal macrophages from BALB/c has been studied. Four extracts (*Bambusa vulgaris, Hura crepitans, Mangifera indica, Simarouba glauca*) were showed the most specific cytotoxic effects on intracellular amastigotes. In addition, the effects of the extracts of different species of Artemisia were placed in the next rank orders (18). A review of the literature on tarragon in addition to several other plants has been done in terms of anti-amastigote effects. Among these, *Aloe Vera* was significantly effective. In a study on the aqueous and methanol extracts of *Aloe secundiflora* in vitro, the extract of the plant can directly stimulate the immune system against the parasite by increasing the amount of nitric oxide in macrophages. For instance, the aqueous extract of this plant significantly (*P*<0.05) reduces macrophages infected with *L. major* amastigotes form. Interestingly however, the methanol extract of this plant did not affect the number of parasites in macrophages. Therefore, in the case of some plants, aqueous extracts are preferable to alcoholic extracts (methanol) (19). Other plants compared with tarragon antileishmanial extract include the use of garlic and onion in the treatment of Leishmanial infections. Substances allicin and di allyl disulfide damage amastogates by stimulating IFN-γ and this way accelerate killing of amastigotes (20).

With regard to the effect of tarragon on *L. major* amastigote form, the results of our research is among the first specialized studies on the impact of tarragon on this parasite. In previous studies, the effects of tarragon on the amastigotes of *L. amazonensis* have been investigated. However, the extract at a concentration of 25 μg/ml did not show a significant effect on the proliferation of promastigotes and amastigotes of the parasites even after the lapse of up to 96h. The extract at a concentration of 25 μg/ml within 24 h of testing has been ineffective up to 98% on the viability of parasite sample (21). Moreover, amistogates, which survive after exposure to the extract and their structures are not damaged, can later be converted to promastigotes (22). However, treatment with 25 μg/ml of this herbal extract does not have any significant effect on the number of amastigotes and promastigotes and their differentiation within 48 h (23). Other plants have already been tested on Leishmania amastigotes and the results were quite different. Although the chemical composition of tarragon has previously been studied (24), the evaluation of the effects of tarragon on the amastigotes of *L. major* is the only specialized research conducted in this field thus far. Resistance to or susceptibility to Leishmania is dependent upon the host immune response to the parasite. Resistance to cellular immunity, the developed Th_1_ response, and the susceptibility to the parasitic infection are caused by Th_2_ immune shift (25).

Th_1_ cells produce cytokines such as IFN-γ and IL_2_ and Th_2_ cells produce IL_4_ and IL_10_ (26).

The results showed that different groups have significant differences in terms of IFN-γ cytokine production. This difference is particularly significant between the groups receiving 20 and 25 mg/ml of the extract and the one without extract (*P*<0.0001).

There are numerous studies conducted on the effects of different species of Artemisia on the immune system. The strengthening effects of *A. Afra* were reflects on macrophages and increased resistance to intracellular bacteria by consuming the extract of Artemisia (27). The effect of *A. dracuncultus* on innate immunity in which the consumption of the plant increased the neutrophils count (28).

The therapeutic effect of *A. dracuncultus* was proven on tuberculosis infection. The results showed increased immunity against creating the granuloma and better control of the infection (29). The anti-inflammatory effects of Artemisia extract. Such findings appear in contrast to our study; however, the only measured cytokines were IL6 and TNF-α in which the anti-inflammatory property may pertain to its effect on innate immunity. Although scant data exists in the literature specifically investigating the effect of *A. dracuncultus* on the production of IFN-γ, studies indicate cellular immune boosting effects of the plant, particularly in combating intracellular microbes. In other words, much of the research is in accordance with our results. In the present study, IFN-γ production was increased as it was added to the concentration of the herbal extract of *A. dracuncultus*.

The reduction of IL-4 production after treatment with *A. dracuncultus* extract was also among other findings. This effect increased with the increasing concentrations of the extract.

These increasing concentrations of the extract also resulted in an increased ratio of IFN-γ/IL-4 that, in fact, represents an increase in Th_1_/Th_2_ response ratio indicating the generation of immune shift towards cellular immunity of Th_1_. A shift in the immune response can contribute to better resistance against leishmanial infection. The findings in the present study showed the immunomodulatory effects of tarragon in HPBMCs.

## Conclusion

By increasing the dose of *A. dracunculus* extract, the levels of IFN-γ and IL-4 gradually increased and declined, respectively.
